# 

**Published:** 1913-03

**Authors:** 


MARCH, 1913.

*Vol. XXXI.* *No.* 119.

### THE BRISTOL MEDICO-CHIRURGICAL JOURNAL

*PUBLISHED UNDER THE AUSPICES OF*

*THE BRISTOL MEDICO-CHIRURGICAL SOCIETY.*

*Editor:* P. WATSON-WILLIAMS, M.D.

WITH WHOM ARE ASSOCIATED

J. MICHELL CLARKE, M.A., M.D., LL.D.,

J. LACY FIRTH, M.S., M.D., JAMES SWAIN, M.S., M.D.

J. A. NIXON, B.A., M.B., *Assistant Editor.*

*Editorial Secretary:* J. M. FORTESCUE-BRICKDALE, M.A., M.D.

BRISTOL: J. W. ARROWSMITH LTD.

London: J. & A. Churchill, 7 Great Marlborough Street.

*Price One Shilling and Sixpence.*

